# Postoperative Excessive Bleeding following Stainless-Steel Crown Placement in Healthy Children with a Suggested Approach for Prevention

**DOI:** 10.1155/2023/6805636

**Published:** 2023-02-08

**Authors:** Liat Oren, Shoshana Spierer, Silvina Friedlander Barenbaum, Noam Yarom, Dan Ben-Amitai, Malka Ashkenazi

**Affiliations:** ^1^Sheba Medical Center, Ramat Gan 52621, Israel; ^2^Sheba Medical Center, Ramat Gan, Israel; Medical School of Medicine, Tel-Aviv University, Tel-Aviv, Israel; ^3^Pediatric Dermatology Unit Schneider Children Medical Center, Petach-Tikva, Israel

## Abstract

Stainless-Steel Crown (SSC) placement is a common treatment in children, usually without significant adverse consequences. The present case series reports six healthy children were admitted to emergency rooms in two medical centers with delayed excessive bleeding from their gingiva, adjacent to newly placed SSCs. The bleeding, in some cases, was so extensive, that it induced vomiting and anxiety, among the children and their parents, and in two cases required surgical suturing. In all six cases, the most probable etiology was a toxic reaction to the released nickel or chromium ions from the SSCs, exacerbated by contact with wounded and bleeding gingiva. Expression of this cytotoxic mode of action, due to metal ions released from SSCs, is not well documented in children. Our aims are to raise awareness of this unique complication and to suggest an approach to minimize and prevent its occurrence. Recommended immediate treatment includes frequent rinsing of the gingiva to wash out released metal ions and decrease their toxic effect. Furthermore, in the following appointments, we recommend using only SSCs previously soaked in water for several weeks, using zirconia crowns, or using Hall's technique when appropriate.

## 1. Introduction

Stainless-Steel Crown (SSC) placement is a common treatment option in primary and young permanent molars with deep caries lesions, pulp treatment, and developmental enamel defects. This treatment is superior to multi-surface fillings and reduces the risk of major failure and pain in the long term [1, 2] with a 97.2%—5-year survival rate [3–5]. Its main adverse consequences include unaesthetic appearance and increased plaque accumulation when adaption is not optimal. Interestingly, its marginal extension does not induce gingivitis [6–10]. Another rare complication described previously in only two cases by Helder et al. is postoperative persistent bleeding several hours after the placement of SSC [11]. The present case series describes this rare complication in additional six healthy children who were referred because of the bleeding to the emergency room (ER)/pediatrician for treatment by six dentists. Our aims are to raise awareness of this unique complication and to suggest treatment approaches to stop the bleeding, minimize and prevent its occurrence during the event, and the following treatments.

## 2. Case Descriptions

### 2.1. Effectively Stopping the Bleeding by Suturing the Gingiva and by Antifibrinolytic Tranexamic Acid

Case 1: A healthy 3-year-old boy presented at the ER at a tertiary Medical Center with excessive bleeding following the placement of SSC. Consequently, his gingiva was sutured under general anesthesia (GA) in the operative room to control the bleeding. A year later he arrived at a dental clinic for the continuation of his treatment. Since the new treatment plan included placement of SSCs, his mother expressed concerns about potential bleeding but was persuaded to accept the offered treatment, given an explanation that the previous bleeding event was probably not triggered by the crown placement. Eighteen hours following the placement of an SSC on his primary upper second molar, heavy gingival bleeding adjacent to the SSC appeared. The patient required hospital readmittance, where again his gingiva was sutured under GA in the operative room to control the bleeding. The results of coagulation tests were found to be within the normal range.

Case 2: A healthy 6-year-old girl arrived for consultation with her dentist and her pediatrician because of excessive gingival bleeding 2 days post-treatment with an SSC, in her primary mandibular first molar. During the same appointment, space maintenance (band and loop) was also placed, to preserve the space of a previously extracted upper first molar. She was treated with a Tab of antifibrinolytic tranexamic acid 500 mg/Tab, 3 times/day (Teva), and the bleeding stopped completely. Coagulation function assessments were not performed despite the family doctor's recommendation.

### 2.2. Effectively Stopping the Bleeding by Continuously Rinsing the Oral Cavity with Water

**Figure 1:**
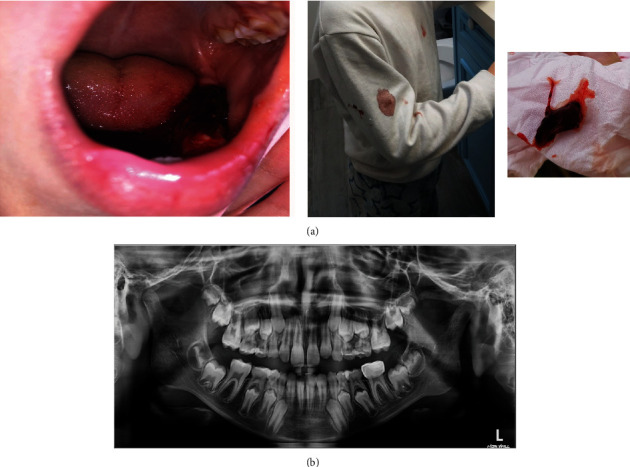
Patient case 3. (a) Bleeding and blood clot 36 hours after placement of SSC on his permanent lower left first molar. (b) Six months postoperatively, no radiographic pathology associated with the SSC on a permanent lower molar.

Case 3: A healthy 7.5-year-old boy with no history of allergy presented to the ER at a tertiary Medical Center with excessive gingival bleeding 2 days after treatment with SSC in his permanent lower first molar. Excessive gingival bleeding was evident around the crown, blood clots covered his SSC, filled his mouth, and were absorbed in his clothes (Figure 1(a)). The child was admitted from ER to the oral surgery department to control his bleeding. Since his bleeding ceased following repeated water rinsing, the child was discharged home with a recommendation to replace the newly placed SSC. The bleeding re-occurred following several hours at home and decreased again following rinsing.

Two days later, the clinical re-evaluation at a private clinic revealed healthy gingiva surrounding the crown with no sign of pathology and no residual cement in the gingival sulcus. Therefore, the pediatric dentist recommended follow-up only. Despite the medical recommendation, allergy tests and a full evaluation of the coagulation functions were not performed due to the parent's objection, mainly because their concerned about their child's fear of needles, and since their child has never suffered from any allergy consequences. Follow-up after 6 months, no recurrent bleeding was reported, and the panoramic X-ray revealed healthy alveolar bone surrounding the treated tooth (Figure 1(b)).

Case 4: A healthy girl aged 4.5 years presented at the ER at our Tertiary Medical Center, with excessive bleeding originating from the gingival sulcus of her primary lower right second molar, 3 days following dental treatment, which included pulpotomy and placement of an SSC. Her dental history revealed that 6 months prior she had received SSC placement (in her primary lower left second molar), yet without any adverse consequences. The girl was very anxious from the unstoppable bleeding. Despite her parent's attempt to stop the bleeding by placing ice popsicles, it resulted in only temporary slightly decreased bleeding.

**Figure 2:**
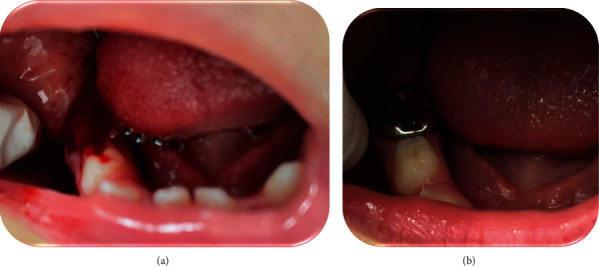
Patient case 4. (a) Excessive bleeding and a blood clot in the mouth of a 4.5-year-old girl 3 days after placing SSCs. (b) The bleeding continued for 18 hours after rinsing the mouth for 30 minutes.

Treatment in ER consisted of local pressure with an oral pack soaked with an antifibrinolytic tranexamic acid solution to stop the bleeding, resulting without improvement. Therefore, she was referred to the pediatric dentistry clinic. On her arrival at our clinic, clinical examination revealed her primary right lower second molar was completely covered with a clot and with active bleeding (Figure 2). The crown was fitted correctly. The girl was instructed to rinse her mouth continuously for 30 minutes with water, which completely stopped the bleeding. The girl was not tested for contact allergy since she had already undergone an SSC placement without any adverse effect. Her coagulation evaluation was within normal range.

### 2.3. Effectively Stopping Future Bleeding by Pre-Soaking the SSCs in Water

**Figure 3:**
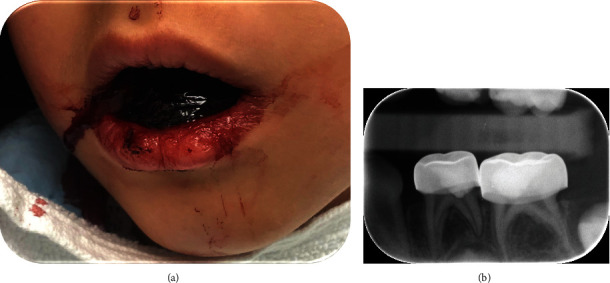
Patient case 5. (a) Excessive bleeding and a blood clot in the mouth of a girl, 16 hours after placing SSCs on her primary lower left first and second molars. (b) A periapical radiograph taken 1-day postoperatively reveals no pathology that may contribute to the excessive bleeding.

Case 5: A healthy 5-year-old girl presented to the ER at our tertiary Medical Center with a blood clot filling her mouth (Figure 3(a)) following re-occurring bleeding at different intensities accompanied by vomiting. The girl received 2 days earlier dental treatment, which included pulpotomies and placement of two SSCs in her primary lower left first and second molar. The bleeding originated from the gingiva surrounding the newly placed SSCs. Since the bleeding continued, the girl was hospitalized for further observation and evaluation in our pediatric dentistry clinic. Clinical examination revealed that the SSCs were with minimal overhanging and with no residual cement in the gingival sulcus. The periapical radiographs of the treated teeth were without pathologic findings (Figure 3(b)), therefore, the gingiva around the SSCs were sutured, under nitrous oxide analgesia, which eventually stopped the bleeding. Her coagulation evaluation was within normal range, and a mild allergy to nickel (+1) was detected.

Since the girl needed to be treated with five more SSCs, and in an assumption that the bleeding was triggered by released metal ions from the SSCs, it was decided to try to prevent the re-occurrence of the postoperative bleeding by soaking the relevant SSCs (all sizes) in water for 4 weeks before use. The next treatment was performed under GA. Two days postoperatively, only a very mild gingival bleeding surrounding the newly placed SSCs appeared but stopped completely following rinsing. No further consequences occurred.

Case 6: A boy aged 4.5 years old, presented at the ER at our tertiary Medical Center in the afternoon with excessive bleeding from the gingival sulcus, 3 days post-treatment, with newly placed SSCs on his primary mandibular right first and second molar. The blood clots covered his SSCs and filled his mouth. Medical history revealed that the child was diagnosed at 1 year of age with mild idiopathic thrombocytopenic purpura (ITP) following a viral infection, but he had never experienced previous excessive bleeding episodes and had never received any treatment. His last routine PLT count, 6 months prior, was 107,000. Before referral to the ER, his dentist sutured the gingiva around the crown, which achieved only a temporary improvement. In ER in an attempt to control the bleeding by pharmacological and mechanical means, the patient received IV antifibrinolytic tranexamic acid at a dosage of 500 mg (Teva) and was asked to bite forcefully to create local pressure with an oral pack soaked with antifibrinolytic tranexamic acid solution 500 mg/5 ml. Nonetheless, although this treatment reduced the bleeding, it did not stop. His blood test showed a PLT count of 150,000, and his coagulation tests were within the normal range (INR = 0.99, PTT = 42). His hemoglobin was lower than 6 months prior (11.9 vs. 12.5 g/dL).

**Figure 4:**
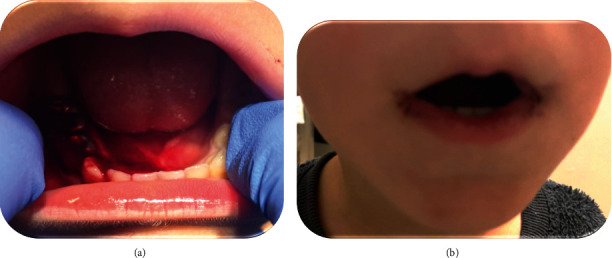
Patient case 6. (a) Excessive bleeding and a blood clot, 4 days after placing 2 SSCs and following receiving IV antifibrinolytic tranexamic acid and biting on an oral pack soaked with solution of antifibrinolytic tranexamic acid. (b) Three and four days after placing 2 SSCs that were pre-soaked in water for 5 weeks before use. Small blood clots were visible, only in the morning, at the corner of the child's lips.

The next morning, the child was admitted to our department of pediatric dentistry for further evaluation. Clinical examination revealed that the child still had blood clots over his crowns, but without active bleeding (Figure 4(a)). Therefore, he was discharged and according to our previous experience, we recommended performing continuous mouth rinsing with water at home, if and when the bleeding resumes. Follow-up by telephone call confirmed that the repeated water-rinsing indeed was effective in eventually completely stopping the bleeding only on the following day at noon.

Since the boy needed two more SSCs in his primary mandibular left first and second molars, we decided to confirm our approach of soaking the crowns in water until the next appointments, to prevent postoperative bleeding. The next treatment was performed 5 weeks later, in which the child received placement of two SSCs. Three and 4 days postoperatively, a small drop of clotted blood was visible in the morning at the corner of his lips (Figure 4(b)). No further consequences occurred. Despite referral to allergy tests for chromium and nickel, the child was not examined.

## 3. Discussion

In the present case series, we describe unusual extensive gingival bleeding following placement of Ion® and Unitek® SSCs in six healthy children. The bleeding in some cases was so extensive that it induced vomiting and anxiety among the children and their parents, and three cases (1, 5, and 6) required surgical suturing of the adjacent gingiva, under GA or nitrous oxide sedation. In one of the cases, the recommendation of the oral surgeon was to replace the SSCs, and in two cases, the pediatric dentist raised the option of replacing the SSC. Yet, no patient was treated by replacing the associated SSC.

In a literature search, only one report of two patients was found, which described a similar consequence following placements of SSC [11]. However, in the present report, we described six patients with varied consequences of delayed excessive bleeding after placement of SSCs and longer follow-up. Three cases (2, 4, and 6) required treatment with antifibrinolytic tranexamic acid via IV, oral Tabs, or locally by soaking an oral pack with the antifibrinolytic tranexamic acid solution and letting the child bite on it. While in two cases (3 and 4), repeated rinsing of the mouth with tap water for 30 minutes was sufficient to control the bleeding. Furthermore, we confirmed the effectiveness of our suggested therapeutic as well as preventive approaches. For prevention of future recurrence, two cases (5 and 6) were treated by SSCs that were pre-washed for 1 month in tap water. In a literature search in the English language (PubMed, Scopus, and Google Scholar), we found *no* optional clinical approach to decrease the release of these metal ions by pre-soaking the SSCs in water before treatment.

The etiology of extensive gingival bleeding is not fully understood, yet several pathological mechanisms were suggested. First, iatrogenic injury to the gingiva during tooth preparation or placement of long SSCs. However, if this was the case, the bleeding would have started immediately following absorbance of the vasoconstrictor (from local anesthesia) into the bloodstream, about 1 hour postoperatively, and not delayed bleeding 1 or 2 days after placing the SSCs as described in this case series. Moreover, the postoperative radiographs which were taken in cases 3 and 5 confirmed that the length of the crowns probably did not contribute to the extensive bleeding (Figures 1 and 3).

A second mechanism might include undiagnosed bleeding disorders. However, in all our cases, the children had never previously suffered from excessive bleeding, nor suffered from any hematologic or genetic disorders (apart from one case). All the boys were circumcised at the age of 8 days, the girl in case 2 underwent extraction of her primary molar, 2 weeks prior to treatment, and the girl in case 5 had a forehead injury the previous month, which left a visible scar; all cases were without any excessive bleeding consequences. Moreover, the patients in cases 1, 3, and 5 underwent full laboratory coagulation evaluations, which were found to be within the normal range. Accordingly, it may be concluded that bleeding disorders were probably not the cause of the extensive bleeding in these cases.

A third mechanism might be a delayed contact allergic reaction to one or more of the metal ions released from the SSCs: nickel, chromium, or iron [12–16]. Nickel is the leading contact allergen in most industrialized countries worldwide, with a prevalence of 8–19% in adults and 8–10% in children and adolescents [17–22]. Contact allergy to nickel in the oral cavity results mainly following exposure to stainless-steel dental alloys, which are widely used in orthodontics, prosthodontics, and pediatric dentistry, in SSCs, and as space maintainers [23]. It was shown that orthodontic appliances containing nickel may produce a burning sensation along with gingivitis, gingival hyperplasia, periodontitis, erythema, and angular cheilitis [12–15]. Furthermore, SSCs were strongly associated with contact allergic dermatitis, oral lichenoid lesions, xerostomia, and metallic taste [16]. Nonetheless, it should be pointed out that in all the described cases, bleeding in the children ceased following treatment, which did not include the removal of the associated SSCs.

Therefore, another more probable mechanism, in the case of excessive bleeding following SSC placement, is possibly a toxic reaction of the injured gingiva to the released metal ions from the SSC. It has been previously established that one of the most common mechanisms of adverse reactions induced by metals (i.e., metal toxicity, sub-toxic reactions) is due to the gradual, continuous release of ions from dental materials, which are then absorbed through oral mucosa into the body. This cytotoxic mode of action was confirmed by both *in vitro* studies evaluating the dynamic of ions released from metal dental appliances, as well as by *in vivo* studies assessing the concentration of the released metal ions in saliva, urine, or hair of children, over time following treatment with metal crowns. *In vitro* studies demonstrated that over time concentrations of metal ions were found potentially capable of altering cellular functions. In one study, it was shown that metal ions can alter the monocyte metabolism and thus may be significant to monocyte function [24]. In another study, nickel ions released from nickel-chromium alloys showed evidence of a high cytotoxic effect on fibroblast cell cultures [25].

An *in vivo* study demonstrated that following the application of conventional and self-ligating brackets, salivary levels of nickel and chromium progressively increased from days 1 to 7 and then decreased on day 30 [26]. Similarly, significant amounts of nickel and chromium were found in the saliva [27] and hair [28] of children post-treatment with SSCs. A prospective longitudinal study on 37 pediatric patients suggested that metal crowns cause genotoxic damage at the cellular level of the oral mucosa, as they found that 45 days after exposure there was a significantly increased in the number of buccal epithelial cells with exposed micronuclei (from 4.67 ± 0.15 to 6.78 ± 0.167/1000 cells *P* < 0.05). Additionally, significantly high urinary concentration levels of nickel were detected (from 2.12 ± 1.23 to 3.86 ± 2.96 mg Ni/g creatinine *P* < 0.05) [29].

In an *in vitro* study, it was shown that substantial amounts of chromium and nickel ions were released over a period of 1–28 days in an artificial saliva medium [30]. Moreover, high absorption of metal ions was found in the cementum of primary molars covered 2 years by SSCs; measuring a significantly (*P* < 0.001) 5- to 6-fold higher concentration of nickel, chromium, and iron ions, as compared with their concentrations in the cementum of intact primary molars [31].

Interestingly, the release of these metal ions from stainless-steel alloys increased in an acidic environment [26, 27]. Of note, many children who suffer from postoperative pain following placement of SSCs avoid toothbrushing for several days post-treatment, which may lead to acidic salivary pH, subsequently inducing an increase in the release of metal ions. Saliva-acidity may indirectly be caused by xerostomia, which can also be induced by these alloys [16]. Corresponding to the aforementioned studies on the dynamic of metal ions released over time from SSCs, leading to high concentrations of metal ions in saliva following treatment, with a potential cytotoxic effect on cellular functions; indeed, in all the children described in the current report, the bleeding appeared 10–72 hours post-treatment, which may indicate a minimum concentration of the released ions to interfere with the coagulation process.

Interestingly, the second case described a girl, who had received during the same treatment setting, a space maintenance appliance, which has a similar composition as the SSCs, yet without a similar consequence. Moreover, case number four received previously SSCs placement without adverse reactions. These may result from the fact that different batches of SSCs release different concentrations of ions, from less injury to the gingival during the operative treatment or from a better practice of oral hygiene immediately after treatment, which subsequently increases the salivary pH. In this regard, it should be pointed out that two of these children were treated 5–7 years ago and the rest during the last 2 years. Moreover, some were treated by Ion® and others by Unitek® SSCs, which may rule out an option of side effects of only one batch of SSCs. Taken together, it can be speculated that the toxic reaction from released metal ions, from the SSCs, might be exacerbated in contact with wounded and bleeding gingiva. Accordingly, another approach to prevent bleeding following the placement of SSCs may include the use of Hall's technique in children who suffer from moderate caries lesions, which is associated with less injury to the gingiva.

Cases 5 and 6 described children who suffered from excessive postoperative bleeding, but after pre-soaking the next SSCs in water, the treatment resulted in only mild gingival bleeding (Figure 4(b)). Moreover, in case 6, rinsing the mouth continuously for 30 minutes ceased the bleeding even though previous treatment with antifibrinolytic tranexamic acid was not effective. This fact reinforces the notion that metal ions released from the SSCs, and contact with the injured gingiva, play an essential role in postoperative excessive bleeding following placement of SSCs. Further studies are recommended to evaluate the amount and types of ions released from different manufacturers of SSCs and the effect of these ions on the coagulation process.

In conclusion, a unique adverse effect of SSCs was presented in this case series. Should excessive bleeding following SSC placement develop, the recommended immediate treatment may include continuous rinsing of the gingiva to wash out the released metal ions and decrease their toxic effect. There is no need to replace these SSCs. Furthermore, in the following appointments, we recommend using SSCs that were previously soaked in water for several weeks, using zirconia crowns, or considering the use of Hall's technique when appropriate.

## References

[1] Seale N. S., Randall R. (2015). The use of stainless-steel crowns: a systematic literature review. *Pediatric Dentistry*.

[2] Innes N. P., Ricketts D., Chong L. Y. (2015). Preformed crowns for decayed primary molar teeth. *Cochrane Database of Systematic Reviews*.

[3] Discepolo K., Sultan M. (2017). Investigation of adult stainless steel crown longevity as an interim restoration in pediatric patients. *International Journal of Paediatric Dentistry*.

[4] Zahdan B. Z., Szabo A., Gonzalez C. D., Okunseri E. M., Okunseri C. E. (2018). Survival rates of stainless-steel crowns and multi-surface composite restorations placed by dental students in a pediatric clinic. *The Journal of Clinical Pediatric Dentistry*.

[5] Hiller M., Roloff T., Kühnisch J., Heinrich-Weltzien R. (2014). Clinical success of stainless-steel crowns placed under general anaesthesia in primary molars: an observational follow up study. *Journal of Dentistry*.

[6] Einwag J. (1984). Effect of entirely preformed stainless-steel crowns on periodontal health in primary, mixed dentitions. *ASDC Journal of Dentistry for Children*.

[7] Prabhu S., Krishnamoorthy S. H., Sathyaprasad S., Sharath C. H., Divyia J., Mohan A. (2018). Gingival, oral hygiene and periodontal status of the teeth restored with stainless steel crown: a prospective study. *Journal of the Indian Society of Pedodontics and Preventive Dentistry*.

[8] Hensten-Pettersen A. (1992). Casting alloys: side-effects. *Advances in Dental Research*.

[9] Durr D. P., Ashrafi M. H., Duncan W. K. (1982). A study of plaque accumulation and gingival health surrounding stainless steel crowns. *ASDC Journal of Dentistry for Children*.

[10] Guelmann M., Matsson L., Bimstein E. (1988). Periodontal health at first permanent molars adjacent to primary molar stainless-steel crowns. *Journal of Clinical Periodontology*.

[11] Helder C., Fleagle J., Alimorad L., Cottam M. (2021). Postoperative bleeding complications after stainless steel crown placement: a case series. *Journal of Clinical Pediatric Dentistry*.

[12] Lamster I., Kalfus D., Steigerwald P., Chasens A. (1987). Rapid loss of alveolar bone association with nonprecious alloy crowns in two patients with Ni hypersensitivity. *Journal of Periodontology*.

[13] Starkjaer L., Menné T. (1990). Nickel allergy and orthodontic treatment. *European Journal of Orthodontics*.

[14] Bishara S., Barrett R., Selim M. (1993). Biodegradation of orthodontic appliances. Part II. changes in the blood level of Ni. *American Journal of Orthodontics and Dentofacial Orthopedics*.

[15] Janson G., Dainesi E., Consolaro A., Woodside D., Freitas M. (1998). Ni hypersensitivity reaction before, during, and after orthodontic therapy. *American Journal of Orthodontics and Dentofacial Orthopedics*.

[16] Muris J., Goossens A., Gonçalo M. (2015). Sensitization to palladium and nickel in Europe and the relationship with oral disease and dental alloys. *Contact Dermatitis*.

[17] Ahlström M. G., Thyssen J. P., Menné T., Johansen J. D. (2017). Prevalence of nickel allergy in Europe following the EU nickel directive—a review. *Contact Dermatitis*.

[18] Diepgen T. L., Ofenloch R. F., Bruze M. (2016). Prevalence of contact allergy in the general population in different European regions. *The British Journal of Dermatology*.

[19] Mortz C. G., Lauritsen J. M., Bindslev-Jensen C., Andersen K. E. (2001). Prevalence of atopic dermatitis, asthma, allergic rhinitis, and hand and contact dermatitis in adolescents. The Odense Adolescence Cohort Study on Atopic Diseases and Dermatitis. *The British Journal of Dermatology*.

[20] Fors R., Persson M., Bergström E., Stenlund H., Stymne B., Stenberg B. (2008). Nickel allergy-prevalence in a population of Swedish youths from patch test and questionnaire data. *Contact Dermatitis*.

[21] Kręcisz B., Chomiczewska D., Palczynski C., Kiec-Swierczynska M. (2012). Contact allergy to metals in adolescents. Nickel release from metal accessories 7 years after the implementation of the EU Nickel Directive in Poland. *Contact Dermatitis*.

[22] Lagrelius M., Wahlgren C. F., Matura M., Kull I., Lidén C. (2016). High prevalence of contact allergy in adolescence: results from the population based BAMSE birth cohort. *Contact Dermatitis*.

[23] Syed M., Chopra R., Sachdev V. (2015). Allergic reactions to dental materials—a systematic review. *Journal of Clinical and Diagnostic Research*.

[24] Wataha J. C., Lockwood P. E., Schedle A., Noda M., Bouillaguet S. (2002). Ag, Cu, Hg and Ni ions alter the metabolism of human monocytes during extended low-dose exposures. *Journal of Oral Rehabilitation*.

[25] Elshahawy W. M., Watanabe I., Kramer P. (2009). *In vitro* cytotoxicity evaluation of elemental ions released from different prosthodontic materials. *Dental Materials*.

[26] Sahoo N., Kailasam V., Padmanabhan S., Chittaranjan A. B. (2011). *In vivo* evaluation of salivary nickel and chromium levels in conventional and self-ligating brackets. *American Journal of Orthodontics and Dentofacial Orthopedics*.

[27] Basir L., Meshki R., Behbudi A., Rakhshan V. (2019). Effects of restoring the primary dentition with stainless-steel crowns on children’s salivary nickel and chromium levels, and the associations with saliva pH: a preliminary before-after clinical trial. *Biological Trace Element Research*.

[28] Kodaira H., Ohno K., Fukase N. (2013). Release and systemic accumulation of heavy metals from preformed crowns used in restoration of primary teeth. *Journal of Oral Science*.

[29] Morán-Martínez J., Monreal-de Luna K. D., Betancourt-Martínez N. D. (2013). Genotoxicity in oral epithelial cells in children caused by nickel in metal crowns. *Genetics and Molecular Research*.

[30] Amanna E. N., Bhat S. S., Hegde S. K. (2019). An *In vitro* evaluation of nickel and chromium release from different commercially available stainless-steel crowns. *Journal of the Indian Society of Pedodontics and Preventive Dentistry*.

[31] Keinan D., Mass E., Zilberman U. (2010). Absorption of nickel, chromium, and iron by the root surface of primary molars covered with stainless steel crowns. *International Journal of Dentistry*.

